# Fetal Head Growth and Head Circumference at Birth in Children of Women with Psychotic Disorders and Population-Based Controls

**DOI:** 10.1093/schbul/sbaf171

**Published:** 2025-09-28

**Authors:** Lisanne A E M van Houtum, Dogukan Koc, Sterna A Grundeman, Hans J Duvekot, Hanan El Marroun, Neeltje E M van Haren, Hilmar H Bijma

**Affiliations:** Department of Child and Adolescent Psychiatry/Psychology, Erasmus MC, University Medical Centre Rotterdam – Sophia, 3015 CN Rotterdam, The Netherlands; Department of Child and Adolescent Psychiatry/Psychology, Erasmus MC, University Medical Centre Rotterdam – Sophia, 3015 CN Rotterdam, The Netherlands; Generation R Study Group, Erasmus University Medical Centre, Erasmus University Rotterdam, 3015 GD Rotterdam, The Netherlands; Department of Obstetrics and Gynecology, Division of Obstetrics and Prenatal Medicine, Erasmus MC, University Medical Centre Rotterdam – Sophia, 3015 GD Rotterdam, The Netherlands; Department of Obstetrics and Gynecology, Division of Obstetrics and Prenatal Medicine, Erasmus MC, University Medical Centre Rotterdam – Sophia, 3015 GD Rotterdam, The Netherlands; Department of Child and Adolescent Psychiatry/Psychology, Erasmus MC, University Medical Centre Rotterdam – Sophia, 3015 CN Rotterdam, The Netherlands; Department of Psychology, Education and Child Studies, Erasmus School of Social and Behavioral Sciences, Erasmus University Rotterdam, 3063 ND Rotterdam, The Netherlands; Department of Child and Adolescent Psychiatry/Psychology, Erasmus MC, University Medical Centre Rotterdam – Sophia, 3015 CN Rotterdam, The Netherlands; Department of Obstetrics and Gynecology, Division of Obstetrics and Prenatal Medicine, Erasmus MC, University Medical Centre Rotterdam – Sophia, 3015 GD Rotterdam, The Netherlands; Department of Care Ethics, University of Humanistic Studies, 3512 HD Utrecht, The Netherlands

**Keywords:** psychosis, schizophrenia, fetal growth, neonatal head size, birth outcomes, familial high-risk

## Abstract

**Background:**

Children of parents with psychotic disorders have a ˃50% increased risk to develop mental health problems, and over 30% have developed severe mental illness by early adulthood. Aberrant brain development may underly this familial risk. We aimed to investigate differences in brain development, reflected in fetal head circumference (HC) growth trajectories and HC at birth, between children of women with psychotic disorders and population-based controls.

**Study Design:**

We collected fetal ultrasonography assessments at 20, 30, and 36 weeks of gestational age (GA) from medical records of *N* = 140 pregnant women having a psychotic disorder diagnosis and their *N* = 168 children. In the Generation R study, ultrasonography assessments were performed in the first, second, and/or third trimester in *N* = 8605 pregnant women and their children. In both groups, HC at birth was measured with measuring tape.

**Study Results:**

Using generalized additive mixed modeling, we observed decreased non-linear fetal HC growth for offspring of women with psychotic disorders vs. controls from 30.7 weeks GA onwards. At birth, no significant difference was observed (*b* = 0.22, *95% CI* [–0.133 to 0.573]), although offspring exposed to maternal psychosis showed more obstetric complications and suboptimal birth outcomes, including lower birthweight (*b* = –136.1, *95% CI* [–229.0 to –43.2]).

**Conclusions:**

This study showed decreased fetal head growth during the third trimester and lower birthweight in children of women with psychotic disorders. Together, these findings highlight potential relevance of altered fetal head growth for later neurodevelopmental outcomes and provide directions for possible underlying mechanisms of risk transmission in psychosis.

## Introduction

Psychotic disorders, such as schizophrenia or schizoaffective disorder, are leading causes of disability worldwide.^1^ Generally, these severe mental illnesses (SMIs, ie, psychotic disorders, bipolar disorder, major depressive disorder) greatly impact a person’s capacity to function in daily life, often requiring ongoing treatment and support.^2^  *Children* born from parents with SMI do have a ˃50% increased risk for developing mental health problems themselves, and over 30% have developed SMI by early adulthood.^3^^,^^4^ For psychosis specifically, children born from parents with psychotic disorders have an eightfold increased risk to develop psychosis.^4^ Next to genetic liability, environmental factors, including periconceptional epigenetic changes and prenatal influence related to maternal illness during pregnancy, for example distress and psychotropic medication use, may impact these offspring’s development already in the fetal period, although the underlying mechanisms are still largely unknown.^5–8^ There is an urgent need to elucidate risk factors and signs of aberrant (brain) development as early as possible to inform interventions aiming to prevent mental illness in offspring at familial high-risk for SMIs and optimize their life-course health.

According to the neurodevelopmental model of psychosis, which was introduced over 35 years ago,^9^^,^^10^ the risk of developing psychosis later in life may be shaped by alterations in early brain development, notably during the fetal period. This also aligns well with the developmental origins of health and disease (DOHaD) framework, positing that parental factors, including mental disorders in pregnancy, impact on the intrauterine environment and influence fetal development and later life health outcomes.^11^ Considerable evidence now indicates that in fact many epidemiological, (epi)genetic, neuroimaging, and environmental factors are related to psychosis,^12–14^ with the fetal period playing an important role.^15^ During this period, brain growth is driven by key neurobiological processes, including neurogenesis, synaptogenesis and myelination, leading to the foundation of crucial structural and functional networks, subsequently increasing brain volume.^16^ For example, there is evidence for an association between poor prenatal maternal mental health and decreased fetal head growth,^5^^,^^17^^,^^18^ suggesting a link between pre-existing mental illness and altered early neurodevelopment in offspring. Interestingly, individuals with schizophrenia often have structural deviations of the brain, including *a smaller* intracranial volume (ICV).^19^^,^^20^ Not only is a lower ICV evident among individuals with psychotic disorders, this is also the case in adolescent offspring.^21^^,^^22^ Therefore, aberrant early brain development may, especially during the fetal period where brain growth is accelerated,^23^ form an essential basis in the onset of SMIs in at-risk offspring.

Head circumference (HC) is a commonly used measurement in fetal neurodevelopmental evaluation and pediatric growth assessment.^24^ Given the positive correlation between HC and infant brain volume,^24–26^ HC can serve as a suitable proxy for fetal and neonatal brain growth and development.^27^ Prior work demonstrated that on average, individuals who went on to develop schizophrenia had a smaller HC *at birth* compared to controls.^28–32^ However, these studies had relatively small sample sizes, which questions the robustness and reliability of these findings. A smaller HC at birth may reflect a smaller ICV seen in individuals with schizophrenia^19^ and in their offspring.^22^ Together, these findings support the idea that smaller HC may be an early indicator of altered neurodevelopment in offspring of parents with SMI. However, to date, data on HC and growth trajectories in populations at-risk for SMIs are lacking. By studying fetal HC growth trajectories and head size at birth in offspring of women with and without psychosis, we will increase our knowledge about possible aberrant brain development in children at-risk for SMIs and provide directions for possible underlying neural mechanisms related to the intergenerational transmission of mental illness.

This study aims to investigate differences in early brain development, reflected in fetal HC growth trajectories and HC at birth, between offspring of women with a psychotic disorder and population-based controls. First, we explore differences in fetal HC growth trajectories between both groups. Second, we investigate if offspring of women with a psychotic disorder have a smaller HC at birth compared to population-based control offspring.^28–32^ We expect a difference in offset, and/or a decreased growth, based on prior findings in HC at birth in individuals with schizophrenia compared to controls.^28–32^

## Method

All study measures, hypotheses, and analyses were preregistered prior to data analyses (https://osf.io/5974s).

### Participants

In this retrospective observational study, we collected data from medical records of *N* = 155 pregnant women who had a diagnosis of a psychotic disorder and a total of *N* = 192 offspring (*n* = 37 siblings). All pregnant women known with a psychotic disorder who were under obstetric care of the department of Obstetrics and Gynecology at Erasmus University Medical Centre in Rotterdam, the Netherlands and delivered between August 2006 and August 2023 were included in the clinical group. All women having a singleton pregnancy were included if they delivered with a live-born infant and had at least one measurement of fetal HC data of their child (ie, ultrasound scans ⁓20, 30, and/or 36 weeks GA). Women were excluded in case of a chromosomal or fetal structural abnormality known to be associated with abnormal HC. Specifically for HC at birth, only women who delivered after 37-weeks GA, and with an HC measurement at birth were included. After exclusions (Supplement 1), the sample consisted of data from *n* = 135 women and their *n* = 161 live-born offspring (*n* = 26 siblings) for the fetal HC analyses, and *n* = 118 women their *n* = 138 live-born offspring (*n* = 20 siblings) for the HC at birth analyses (total *n* = 140 women and their *n* = 168 live-born offspring, see Table 1). The Medical Ethics Committee of Erasmus Medical Centre declared this study exempt from the Medical Research Involving Human Subjects Act (Protocol no. MEC-2020-0852).

**Table 1:** Participants’ Demographics and Descriptive Statistics

	**Clinical group (n = 168 offspring of n = 140 women)**		**Control group (n = 8605 offspring of n = 8605 women)**		**Between groups** **t-test/U-test** ^**a**^ **/χ** ^**2**^ **-test**
**Variables**	**Mean (SD)/n (%)**	**Range**	**Mean (SD)/n (%)**	**Range**	
**Maternal characteristics**					
** Age (years)**	33.3 (5.91)	16.8–45.0	30.1 (5.31)	15.6–46.9	*U* = 501 504, ***P* <.001**
** BMI**	27.8 (6.18)	16.6–51.9	24.9 (4.55)	15.2–51.2	*U* = 460 205, ***P* < .001**
** Low SES**	26 (15.7%)	–	1786 (20.8%)	–	χ^2^(1) = 2.31, *P* = .129
** Nulliparous, n**	79 (47.0%)	–	4733 (56.7%)	–	χ^2^(1) = 4.67, ***P* = .031**
** Psychotic disorder**					
Schizophrenia or schizophreniform disorder	56 (33.3%)	–	Not assessed	–	
Schizoaffective disorder	27 (16.1%)	–	Not assessed	–	
Brief psychotic disorder	10 (5.95%)	–	Not assessed	–	
Unspecified/other Schizophrenia-spectrum and other psychotic disorder	75 (44.6%)	–	Not assessed	–	
** Psychopathology based on self-reported vignettes**					
Depression	Not assessed	–	1228 (14.3%)	–	
Mania	Not assessed	–	412 (4.79%)	–	
Anxiety	Not assessed	–	686 (7.97%)	–	
Psychosis	Not assessed	–	73 (0.85%)	–	
Anorexia	Not assessed	–	301 (3.50%)	–	
Bulimia	Not assessed	–	387 (4.50%)	–	
Substance use disorder	Not assessed	–	130 (1.51%)	–	
** Psychotropic medication use**	136 (80.9%)	–	237 (3.01%)	–	χ^2^(1) = 2240.2, ***P* < .001**
Antipsychotics	132 (78.6%)	–	9 (0.13%)	–	
SSRIs/SNRIs	17 (10.1%)	–	99 (1.26%)	–	
Benzodiazepine	17 (10.1%)	–	119 (1.52%)	–	
Lithium	8 (4.76%)	–	0 (0.00%)	–	
Anti-epileptics	1 (0.06%)	–	10 (0.14%)	–	
** Substance use**	20 (12.1%)	–	767 (9.88%)	–	χ^2^(1) = 0.67, *P* = .413
Cannabis	17 (10.3%)	–	225 (3.05%)	–	
Other drugs	8 (4.85%)	–	42 (0.56%)	–	
Alcohol	5 (3.03%)	–	560 (7.29%)	–	
** Smoking during pregnancy**	61 (37.0%)	–	1402 (18.9%)	–	χ^2^(1) = 33.9, ***P* < .001**
** Gestational diabetes**	21 (12.9%)	–	89 (1.08%)	–	χ^2^(1) = 163.6, ***P*<.001**
**Child characteristics**					
** Sex, n boys**	78 (46.7%)	–	4336 (50.4%)	–	χ^2^(1) = 0.76, *P* = .385
** Gestational age at ultrasound**					
20 weeks	20.5 (1.19)	18.6–27.0	20.7 (1.19)	18.0–25.0	*U* = 564 414, ***P* = .005**
30 weeks	30.5 (1.20)	26.9–34.3	30.4 (1.05)	25.0–33.9	*U* = 515 569, *P* = .858
36 weeks	35.9 (0.89)	33.9–39.0	35.1 (1.17)	33.9–38.9	*U* = 2508, ***P* < .001**
** Fetal growth restriction** ^**b**^	8 (4.76%)	–	133 (1.61%)	–	χ^2^(1) = 8.08, ***P* = .004**
** 20-week fetal parameters**					
HC (mm)	178.0 (15.3)	154.7–254.0	179.5 (14.8)	136.0–247.0	*U* = 545 288, ***P* = .036**
AC (mm)	158.1 (14.2)	131.9–215.0	157.0 (15.1)	111.5–225.0	*U* = 471 912, *P* = .539
HC/AC ratio	1.13 (0.05)	1.03–1.25	1.15 (0.06)	0.89–1.62	*U* = 588 037, ***P* < .001**
Biparietal diameter (mm)	49.8 (4.42)	42.5–70.0	50.6 (4.24)	38.0–69.0	*U* = 574 623, ***P* < .001**
Femur length (mm)	32.7 (3.30)	27.1–48.0	33.5 (3.66)	21.0–53.2	*U* = 568 710, ***P* = .003**
Trans-cerebellar diameter (mm)	20.8 (1.64)	18.1–30.7	21.2 (1.88)	15.6–30.0	*U* = 426 147, ***P* = .029**
Estimated fetal weight (g)	373.2 (92.1)	266.0–917.0	382.9 (96.3)	168.7–1037.7	*U* = 419 504, *P* = .256
**Birth outcome (any GA)**					
** Gestational age at birth**	38.9 (2.20)	28.6–42.6	39.8 (1.90)	20.9–43.7	*U* = 930 123, ***P* < .001**
** Preterm birth (<37 weeks)**	18 (10.7%)	–	459 (5.34%)	–	χ^2^(1) = 8.25, ***P* = .004**
** Delivery mode, n vaginal**	118 (70.7%)	–	6816 (87.8%)	–	χ^2^(1) = 42.3, ***P* < .001**
** Head circumference (cm)**	33.9 (2.13)	27.0–39.5	33.8 (1.67)	29.0–39.0	*U* = 328 728, *P* = .380
** Birthweight (g)**	3209.9 (603.9)	1450–4570	3410.8 (561.2)	635–5310	*U* = 851 073, ***P* < .001**
** HC/birthweight ratio**	0.011 (0.0018)	0.0079–0.020	0.0099 (0.0012)	0.0066–0.019	*U* = 242 597, ***P* < .001**
**Birth outcome (GA ≥ 37 weeks)**	*n* = 150		*n* = 8146		
** Gestational age at birth**	39.5 (1.27)	37.0–42.6	40.1 (1.26)	37.0–43.7	*t*(154.4) = 5.97, ***P* < .001**
** Delivery mode, n vaginal**	107 (71.8%)	–	6505 (88.6%)	–	χ^2^(1) = 38.0, ***P* < .001**
** Head circumference (cm)**	34.1 (1.92)	29.0–39.5	33.8 (1.65)	29.0–39.0	*U* = 281 145, *P* = .087
** Birthweight (g)**	3286.9 (505.5)	1985–4570	3486.0 (496.7)	1390–5310	*t*(154.4) = 4.35, ***P* < .001**
** HC/birthweight ratio**	0.010 (0.0013)	0.0081–0.015	0.0099 (0.0012)	0.0066–0.016	*U* = 221 356, ***P* < .001**

^a^As assumptions of normality and/or equal variances were not met, a nonparametric Mann–Whitney U-test was conducted. ^b^Fetal growth restriction was defined as AC or EFW < p10 in the clinical cohort and as decrease in EFW growth between the second trimester and birth of ≥40 percentiles in the population-based cohort.

Abbreviations: AC, abdominal circumference; EFW, estimated fetal weight; GA, gestational age; HC, head circumference.

As a control population, pregnant women (*N* = 9778) and their offspring (*N* = 9778) participating in the Generation R study, a prospective population-based cohort from early fetal life onwards in Rotterdam, the Netherlands (delivery dates: April 2002–January 2006), were included.^33^ All women with a singleton pregnancy, whom delivered with a live-born infant, and with at least one measurement of fetal HC data of their child (ie, ultrasound scans in the first, second, and/or third trimester) were included in the control group. Specifically for HC at birth, only women who delivered after 37-weeks GA, and with an HC measurement at birth were included. The final sample consists of data from *n* = 8518 women and their *n* = 8518 live-born offspring for fetal HC analyses, and *n* = 4452 for HC at birth analyses (total *n* = 8605 women and their *n* = 8605 live-born offspring, see Table 1). The Generation R study was conducted in accordance with the guidelines proposed in the World Medical Association Declaration of Helsinki and was approved by the Medical Ethics Committee of Erasmus Medical Centre. Written consent was obtained from all participants.

### Psychopathology

In the clinical group, information on psychiatric diagnosis, established by a psychiatrist based on Diagnostic and Statistical Manual of Mental Disorders (DSM) criteria, was collected via the medical records. We included all women with schizophrenia, schizoaffective disorder, brief psychotic disorder, or psychotic disorder not otherwise specified (PNOS). For population-based controls, psychiatric symptoms were assessed via self-reported vignettes, see also Enthoven et al.^34^ In particular, women were categorized with a psychiatric disorder if they reported they had suffered from depression, mania, anxiety, psychosis, anorexia, bulimia and/or substance use disorder in their lifetime.

### Fetal and Birth Measurements

For the clinical group, all women were offered a fetal anomaly scan at ⁓20-weeks GA from 2007 onwards. Ultrasound measurements of fetal growth were done at ⁓30- and 36-weeks GA as part of comprehensive antenatal care. For the control group, fetal ultrasonography measurements were performed in the first, second and/or third trimesters.^35^ For both groups, these measurements were conducted by qualified sonographers using standardized procedures according to international quality standards set by the International Society of Ultrasound in Obstetrics and Gynecology.^36^ The records of these ultrasound scans were evaluated to estimate fetal HC. For harmonization purposes, we deviated from the preregistration (https://osf.io/5974s): instead of using HC data from all three trimesters, we only included HC data from the second and third trimesters, as no data from the first trimester was available in the clinical group (although in- or excluding first trimester data did not impact our findings, see Supplement 2). Furthermore, we created a 36-week GA variable for the control group by selecting all women having an ultrasound at ≥33 + 6 weeks GA. The birth parameters HC and birthweight were collected from the medical records. HC at birth was measured by an obstetric or neonatal nurse using a measuring tape.

Next to HC, the following fetal biometric parameters were collected: abdominal circumference (AC), bi-parietal diameter (BPD), and femur length (FL). Additionally, ⁓20 weeks GA trans-cerebellar diameter (TCD) was measured. Estimated fetal weight (EFW) was calculated based on the Hadlock-formula.^37^ In the current study, AC, BPD, FL, TCD, and EFW were only used for descriptive statistics. In both groups, GA was assessed in the first trimester based on crown to rump length of the fetus, and in the second trimester based on BPD. For the control group, both the intra-observer and interobserver reliabilities of fetal biometry in early pregnancy were excellent, with all intraclass correlation coefficients >0.98.^38^ For the clinical group, no data on intra- and interobserver reliabilities were available.

### Confounding Variables

Based on prior studies on fetal growth,^5^^,^^27^ we included child’s sex, maternal age, body mass index (BMI), neighborhood socioeconomic status (SES), parity, smoking, gestational diabetes, substance use, and psychotropic medication use (ie, any exposure during pregnancy), and additionally for HC *at birth* GA at birth and delivery mode, as potential confounders. For the clinical group, these variables were collected from the medical charts. Neighborhood SES was estimated based on postal code, using the corresponding SES based on welfare, education, and labor (SES-WOA) score calculated by Centraal Bureau voor Statistiek (Dutch Bureau of Statistics). This score is calculated based on three characteristics: financial welfare (the national wealth decile of the household), education (the education level of the household) and work (the household’s recent employment history). The average SES-WOA score per neighborhood was calculated based on the sum of the three abovementioned sub-scores per household, not including students. We used publicly available lists from Centraal Bureau voor Statistiek of the average SES-WOA score per postal code area of the year the child was born. As the SES-WOA score is available since 2014, we used the 2014 list to estimate neighborhood SES for all children born before 2014. We divided the participants’ SES-WOA scores in five quintiles, and designated the lowest quintile (ie, SES-WOA score ≤ –0.547) as low SES as opposed to the four other quintiles, to create a low SES variable (yes/no).

For the control group, BMI was measured during the first Generation R study visit. Maternal age, postal code to estimate neighborhood SES, and obstetric information were based on self-report. Maternal prenatal smoking, alcohol, and hard drugs (ie, cocaine, ecstasy, heroin, and other drugs) use was obtained by questionnaires in each trimester. Information on cannabis use during pregnancy was obtained by a questionnaire in early pregnancy and by urine samples. Information on psychotropic medication use during pregnancy was collected via both questionnaires in each trimester and pharmacy records.^17^ To harmonize variables with data from the clinical group, we dummy coded these variables into “not during pregnancy” and “during pregnancy”. Furthermore, we merged prenatal alcohol, cannabis, and hard drugs use to create a single substance use variable (yes/no). Use of antipsychotics, lithium, selective serotonin reuptake inhibitors (SSRIs), serotonin-norepinephrine reuptake inhibitors (SNRIs), benzodiazepine, and/or anti-epileptics were merged to create a single psychotropic medication use variable (yes/no). Birth measurements, ie, GA at birth and delivery mode, were collected from the medical charts.

### Statistical Analyses

Statistical analyses were performed with R (v4.3.2, https://www.R-project.org) using the packages Multivariate Imputation by Chained Equations (MICE), mgcv, gratia, and lme4. Missing data of covariates, ie, child’s sex (0.05%), maternal age (0.01%), BMI (0.90%), neighborhood SES (0.18%), parity (1.22%), smoking (12.5%), gestational diabetes (4.10%), substance use (9.67%), psychotropic medication use (ie, any exposure during pregnancy; 8.39%), GA at birth (0.06%) and delivery mode (9.62%) were imputed using the MICE algorithm.^39^ MICE uses the fully conditional specification method, modeling each variable with missing data as a function of all other variables in the dataset. We generated 25 imputed data sets. Outcome and predictor variables were not imputed. Subsequently, analyses were performed on each completed dataset separately and combined to one pooled estimate.

To analyze how fetal head growth over the different time-points (GA range high-risk group: 18.6–39.0 weeks; population based controls: 18.0–38.9 weeks) varied as a function of group, controlling for covariates, and family membership, we used Generalized Additive Mixed Models (GAMM).^40^ This technique is well suited for fitting nonlinear relationships through local smoothing effects, independent of any predefined model. To counteract potential overfitting and ensure stable estimation of smooth age trajectories, we selected the number of basis smooth functions (k parameters) as four, in line with recommendations from simulation-based studies and applied GAM literature.^41–44^ To model potential non-linear trajectories of fetal head growth, we applied a smoothing function to model GA in weeks (predictor). Opting for smooth splines, we aimed to better capture essential nonlinear changes that traditional polynomials might overlook. We designated fetal HC as the response variable in the GAMM analysis. We also included a smoothing function *f*, incorporating random effects for each subject. Additionally, group membership was considered as a predictor in our GAMM. Furthermore, we incorporated the smooth GA^*^group interaction term in the GAMM to estimate the average change in head growth per gestational week for each group separately.

Trajectories were visually compared across groups with a difference curve. Trajectories were interpreted as significantly different if zero was not included in the confidence interval of the difference curve. Statistically, trajectories were compared using linear mixed effects (LME) models, by assessing whether the change in head growth over time differed significantly between the groups, ie, a significant interaction between group and GA time point, as was done before by Chan et al. (2024).^45^

To examine differences in HC at birth, HC at birth/birthweight, and birthweight between groups, while controlling for our covariates, we used LME models, with offspring HC(/birthweight) as outcome, including family membership as random effect.

A *P*-value of <.05 was considered to be statistically significant in all analyses, and the “false discovery rate” (FDR; Benjamín-Hochberg) correction was applied to correct for multiple testing with regards to the analyses at birth (*n* = 3 outcomes, ie: HC at birth, HC at birth/birthweight, and birthweight).

### Sensitivity Analyses

To assess the robustness of our main analysis, we performed several separate sensitivity analyses with subsets of the control group: (i) excluding women who self-reported having a psychiatric disorder and/or used medication; (ii) retaining women who self-reported having a psychiatric disorder, but excluding women who used medication; (iii) retaining women who used medication, but excluding women who self-reported having a psychiatric disorder (which deviated from our preregistration (https://osf.io/5974s), where we accidentally stated; (iv) retaining women who self-reported having a psychiatric disorder and/or who used medication). Furthermore, we conducted a whole-sample sensitivity analysis excluding offspring with fetal growth restriction and/or offspring of women who used substances (ie, alcohol, hard drugs, cannabis, cigarettes) during pregnancy, given the known negative impact on fetal (head) growth.^46^^,^^47^

## Results

### Descriptive Characteristics

Descriptive characteristics are shown in Table 1. On average, maternal age (*P*<.001) and BMI (*P*<.001) were higher in the clinical group compared to the control group. Psychotropic medication use during pregnancy was, as expected, far more common in the clinical group than in the control group (80.9% vs. 3.0%; *P*<.001), as was having gestational diabetes (12.9% vs. 1.1%; *P*<.001). A higher percentage of women in the clinical group smoked during pregnancy (37.0% vs. 18.6%; *P*<.001), while substance use did not differ between groups (*P*=.413). In the control group, more women were nulliparous vs. the clinical group (*P*=.031). With regards of the child, the distribution of sex did not differ between both groups (*P*=.385). Intrauterine fetal growth restriction was more common in the clinical group vs. the control group (4.8% vs. 1.6%; *P*=.004). Gestational age at birth (*P*<.001), and birthweight (*P*<.001) were on average lower in the clinical group compared to the control group. Furthermore, premature birth (*P*=.004) and cesarean section as opposed to vaginal delivery (*P*<.001), were more common in the clinical group.

### Fetal HC Growth Trajectories

**Figure 1:**
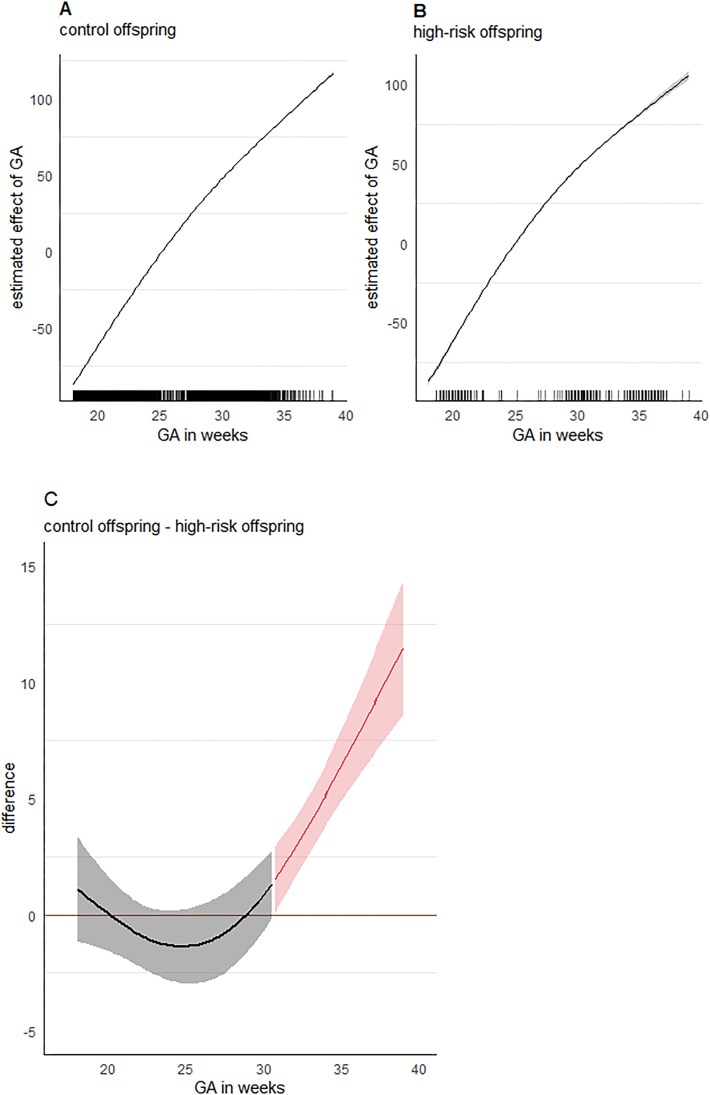
Fetal head circumference growth trajectories using GAMM. Trajectories estimating the effect of gestational age (GA) in weeks for control offspring (A) and offspring of women with a psychotic disorder (B) separately. The y-axis displays the GAM-estimated additive effect of GA in weeks. Markings on the x-axis show individual GA data points. (C) Difference curve graphically showing the differences between the two trajectories in panels A and B (ie, subtracting the estimated effects of GA: Control offspring—High-risk offspring). Trajectories are considered to be significantly different if the confidence interval does not include zero (ie, second part of curve: From 30.7 weeks onwards).

Using GAMM, we observed a non-linear increase in fetal head growth for both the control group (e.d.f. = 1.999, *F* = 380 071, *P*<.001) (Figure 1A) and the clinical group (e.d.f. = 1.996, *F* = 10 437, *P*<.001) (Figure 1B). To compare these trajectories with each other, we plotted a difference curve. The difference curve showed a positive value (a less pronounced increase for offspring of women with a psychotic disorder relative to control offspring) from 30.7 weeks GA onwards, see Figure 1C.

**Figure 2:**
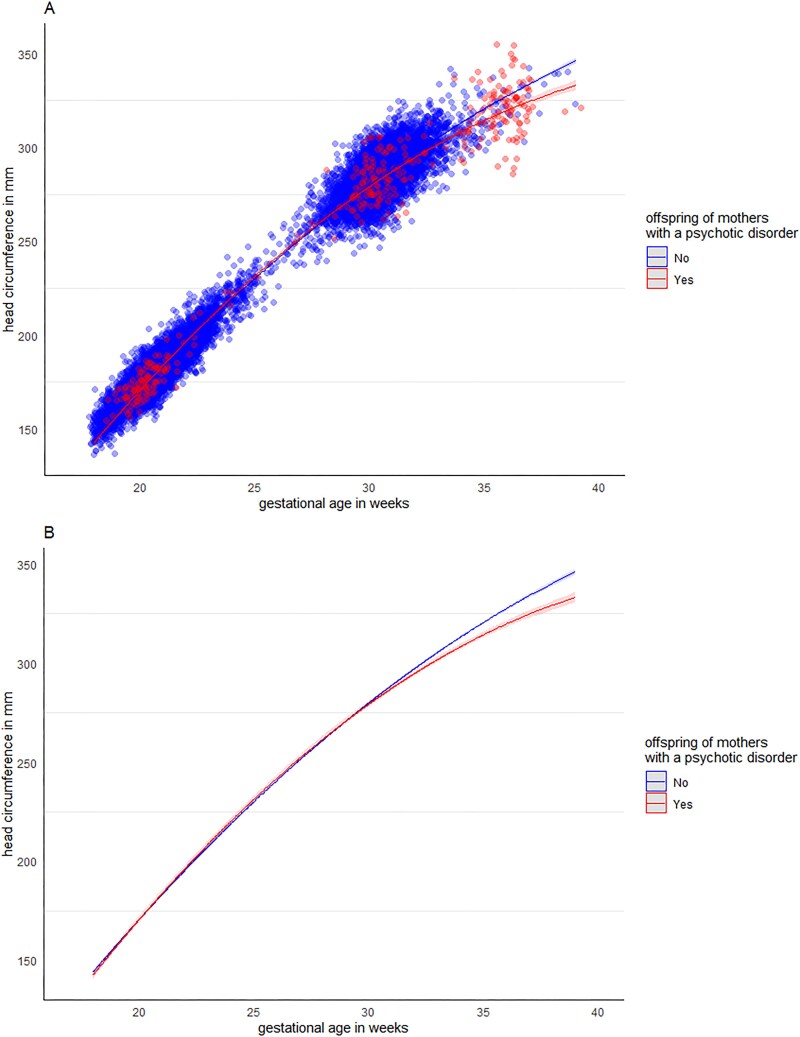
Differences in fetal head circumference growth between offspring of women with psychotic disorders and control offspring, plotted by gestational age in weeks with (A) and without (B) individual data points. Based on linear mixed effects models that were adjusted for child’s sex, maternal age, BMI, SES (based on postal code), parity, smoking, gestational diabetes, substance use, and psychotropic medication use. Shaded areas represent 2.5% and 97.5% confidence intervals (ie, ±1.96^*^standard error).

To test this difference statistically, we used LME modeling, and found a significant difference in the nonlinear trajectory of fetal HC growth between groups, ie, a significant interaction between group and GA^2^ (*b* = –0.07, *SE* = 0.01, *t*(16547.1) = –5.06, *P*<.001, *95% CI* [–0.09 to 0.04]), see Figure 2 and Table 2 for effect parameters, and see Supplement 3 for the model effect parameters when only including main effects. Post-hoc analyses using pairwise comparisons of estimated marginal means with multiple comparison adjustments (Tukey’s HSD) showed that from 31 weeks GA onwards, offspring of women with a psychotic disorder showed decreased fetal HC growth relative to control offspring (*b* = 1.54, *SE* = 0.76, *t*(2323) = 2.04, *P*=.042, *95% CI* [0.05–3.03]; at 32 weeks GA: (*b* = 2.50, *SE* = 0.73, *t*(1987) = 3.42, *P*<.001, *95% CI* [1.07–3.93]).

**Table 2:** Linear Mixed Effects Model Results of Fetal Head Growth Differences Between Offspring of Women with a Psychotic Disorder and Control Offspring

**Effect**	**Estimate b**	**Standard Error**	**Statistic t (df)**	***P*-value**
Intercept	–165.8	3.01	–55.1 (16 534.2)	<.001^***^
Group	–39.5	9.74	–4.06 (16 543.1)	<.001^***^
Gestational age	20.4	0.24	85.0 (16 546.2)	<.001^***^
Gestational age^2^	–0.19	0.00	–40.2 (16 546.2)	<.001^***^
Gestational age x Group	3.33	0.74	4.50 (16 547.2)	<.001^***^
Gestational age^2^x Group	–0.07	0.01	–5.06 (16 547.1)	<.001^***^

*Note.* Findings from linear mixed effects (LME) model testing for a significant difference in the nonlinear trajectory of fetal head growth between offspring of women with a psychotic disorder and control offspring. Pooled LME model effect parameters of fetal head circumference predicted by group (reference = control group), gestational age,^2^ and their interactions are shown. The model was adjusted for child’s sex, maternal age, BMI, SES (based on postal code), parity, smoking, gestational diabetes, substance use, and psychotropic medication use.

### HC at Birth

**Figure 3:**
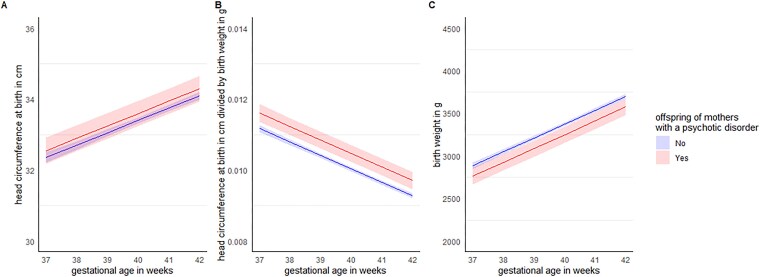
Differences in birth measurements between offspring of women with psychotic disorders and control offspring, plotted by gestational age at birth in weeks. Based on linear mixed models that were adjusted for child’s sex, maternal age, BMI, SES (based on postal code), parity, smoking, gestational diabetes, substance use, psychotropic medication use, GA at birth and delivery mode. (A) Head circumference at birth (in cm) did not differ between groups. (B) The head circumference at birth/birthweight ratio was significantly higher in high-risk offspring vs. controls. (C) Birthweight (in g) was significantly lower in high-risk offspring vs. controls.

At birth, we found no significant difference in HC between groups (*b* = 0.22, *SE* = 0.18, *t*(3899.4) = 1.27, *P*=.205, FDR-corrected *P*=.205, *95% CI* [–0.133 to 0.573]), see Figure 3A. However, we found a significant effect of group on HC/birthweight ratio (*b* = 0.00, *SE* = 0.00, *t*(3055.3) = 4.04, *P*<.001, FDR-corrected *P*<.001, 95% *CI* [0.0003–0.0007]), see Figure 3B, as well as on birthweight (*b* = –136.1, *SE* = 47.4, *t*(3124.8) = –2.87, *P*=.004, FDR-corrected *P*=.006, 95% *CI* [–229.0 to 43.2]), see Figure 3C. HC divided by birthweight reflects the balance between head size and overall body size. That is, HC at birth of offspring of women with a psychotic disorder as compared to control offspring is larger relative to offspring’s overall body size, while total body size is *smaller* in high-risk offspring.

### Sensitivity Analyses

All group findings with regards to fetal HC remained stable after (i) excluding offspring of women who self-reported having a psychiatric disorder and/or used medication within the control group (*n =* 2722 exclusions); (ii) retaining offspring of women who self-reported having a psychiatric disorder, but excluding offspring of women who used medication within the control group (*n =* 973 exclusions); (iii) retaining offspring of women who used medication, but excluding offspring of women who self-reported having a psychiatric disorder within the control group (*n =* 1974 exclusions); (iv) excluding offspring of women with fetal growth restriction and/or who used substances during pregnancy (control group: *n =* 3272 exclusions clinical group: *n =* 70 exclusions), see Supplement 4 for details.

At birth, we found a significant effect of group on HC (*b* = 0.51, *SE* = 0.23, *t*(2905.5) = 2.18, *P*=.029) when rerunning analyses with subgroup iv, indicating that HC at birth was *larger* in offspring of women with psychotic disorders compared to control offspring when excluding offspring with fetal growth restriction and/or offspring of women using substances during pregnancy. The group findings related to HC/birthweight ratio and birthweight remained stable. Furthermore, our main findings did not change when rerunning analyses with subgroup (iii) (ie, retaining offspring of women who used medication, but excluding offspring of women who self-reported having a psychiatric disorder within the control group). However, group findings *disappeared* when rerunning analyses with subgroup (i) (ie, excluding offspring of women who self-reported having a psychiatric disorder and/or used medication within the control group) and subgroup (ii) (ie, retaining offspring of women who self-reported having a psychiatric disorder, but excluding offspring of women who used medication within the control group, see Supplement 5.

Next to our preregistered sensitivity analyses, we explored sex specific effects on HC, see Supplement 6.

## Discussion

This study examined differences in fetal head growth trajectories and HC at birth between offspring of women with a psychotic disorder and population-based controls. We found decreased fetal head growth during the third trimester, ie, after 31 weeks of gestational age, in offspring at familial risk for SMIs compared to control offspring born at term (gestational age ≥ 37 weeks). This finding was robust, as excluding offspring of women who self-reported having a psychiatric disorder and/or used medication in the control group did not affect this finding, neither did excluding offspring of women who used substances or who experienced fetal growth restriction. Although we did not find a difference in HC *at birth* between the groups, birthweight was lower in familial high-risk offspring, even after correction for confounders. Furthermore, HC at birth in these children relative to their overall body size was *larger* compared to controls. This might indicate that brain growth is preserved as long as possible when fetal growth is reduced, but the association could also be primarily driven by birthweight. However, these at birth findings were less robust to our sensitivity analyses, and need to be interpreted cautiously.

Our finding of decreased fetal head growth in the third trimester in maternal high-risk compared to control offspring hints toward an association with brain differences already during the fetal period, possibly putting these offspring at-risk for SMIs later in life. This idea corroborates with the neurodevelopmental model of psychosis,^9^^,^^10^ as well as with the DOHaD framework,^11^ reflecting the importance of the fetal period for lasting consequences across the life course. Furthermore, it aligns with prior work showing that poor prenatal maternal mental health relates to decreased fetal head growth,^5^^,^^17^^,^^18^ and work showing that adolescent *offspring* of people with schizophrenia and adult patients with schizophrenia have a smaller ICV.^19–22^ Especially the deviation from the third trimester onwards is striking, given that during that period myelination starts and neural circuit refinement occurs via processes including synaptogenesis, axonal growth, and pruning, resulting in the earliest structural and functional brain networks.^48^^,^^49^ Therefore, the establishment of neural networks in the early developing brain may be affected in at-risk offspring, under influence of (epi-)genetic factors, in utero exposures, including exposure to increased cortisol levels, medication, as well as other factors, such as low SES, poor nutrition and exposure to substances.^49^^,^^50^ Interestingly, the effect on fetal HC was found *after* correcting for some of these important factors, including low SES, medication and substance use. Further understanding of the factors and mechanisms involved with altered fetal brain development may open up possibilities for early interventions improving outcomes in offspring of women with psychotic disorders.^51^

At birth, however, we did not find a difference in HC between groups. Generally, inferences based on HC at birth are much more imprecise than fetal HC inferences, given both the type of measurement (ie, using a measuring tape instead of ultrasound), and the malleable character of the head during delivery. That is, a vaginal delivery, especially if induced with vacuum extraction, can hugely impact head shape.^52^ Indeed, vaginal delivery, as opposed to cesarean delivery was also a highly significant predictor of HC in our model, whereas it was an insignificant predictor of birthweight, which may question the reliability of HC at birth. Of note, given that the found differences in fetal HC growth are still relatively small, ie, ~13 mm at 39 weeks GA, both the head deformations and the impreciseness of measurement tape may overshadow these subtle differences at birth. Even with ultrasound, there is an intrinsic limitation of measuring head size during the last part of the third trimester, due to the engagement of the fetal head into the pelvis.^53^ Future studies should look at HC trajectories *after* birth, for example after one week and up, and/or use more precise ways to measure postnatal HC, for example via 3D laser scanning or MRI,^54^ to examine whether decreased head growth is (still) present in the postnatal period, and if so, whether or not these children experience catch-up growth over time. Furthermore, given the exceptionally rapid brain growth during the earliest days of life, fetuses (and infants) might be particularly vulnerable, but at the same time particularly responsive to interventions,^51^ advocating for the need to both monitor and research families with at-risk offspring in this precarious life phase.

Generally, women with psychotic disorders experienced more obstetric complications and had a suboptimal birth outcome, including gestational diabetes, restricted fetal growth, lower birthweight, lower gestational age at birth, more preterm birth and more cesarean section as opposed to vaginal delivery. Our findings corroborate with those from a recent meta-analysis investigating adverse obstetric and neonatal outcomes in women with schizophrenia-spectrum disorders.^55^ However, this concurrent population-based cohort study revealed that the elevated risk of negative obstetric and neonatal outcomes might largely be explained by maternal confounding factors associated with these disorders, such as maternal substance use (although this did not impact main findings in our study), psychiatric and physical comorbidities, and psychotropic medication use during pregnancy, rather than the disorder itself. Therefore, close monitoring and patient-centered care interventions targeting such modifiable maternal risk factors that may impact fetal brain development are needed to decrease adverse outcomes in women with psychotic disorders and improve their offsprings health across the life course.^55^

A major strength of this study is the number of offspring of women with a psychotic disorder included, being compared with a population-based sample, facilitated by the Generation R study, which further increased the power of this study and the robustness of our findings. However, this study is not without limitations. First, with respect to the clinical group involving women with a psychotic disorder, we could not disentangle the effects of psychotropic medication use on fetal head growth trajectories, given its high incidence. Furthermore, we were not able to assess information on the fathers of these offspring, and we lacked information on the severity of the disorder, nutrition, psychosocial factors, maternal viral infection including COVID-19 infection, periconceptional factors, and experienced stress, which all could have impacted our findings. Particularly COVID-19 infection has previously been associated with pregnancy and neonatal outcomes, including increased maternal stress, more severe maternal illness, offspring gestational age, birthweight, and potentially fetal brain development.^56–58^ Additionally, as preterm birth occurred significantly more often in women with psychotic disorders, this may have introduced a sampling bias. With respect to the Generation R study sample, it should be noted that late third trimester measurements (⁓36 weeks GA) were scarce (ie, *n* = 88), indicating that replication of our findings in larger samples is warranted. Overall, given that multiple measures at multiple timepoints during pregnancy are involved, this study might be especially prone to selection bias. Finally, residual confounding, ie, unmeasured factors associated with both (fetal) head growth and having familial high-risk for SMIs cannot be ruled out due to the observational nature of the study, neither can causality be inferred.

In sum, we found decreased fetal head growth in offspring of women with psychotic disorders compared to controls in the third trimester, ie, from 31 weeks of gestational age onwards. Moreover, these offspring more often had unfavorable birth outcomes, including lower birthweight, more preterm birth and their mothers experienced more obstetric complications. Together, these findings give us directions for possible underlying (neural) mechanisms already during the fetal period related to the intergenerational transmission of risk for mental illness.

## Supplementary Material

Supplementary_materials_sbaf171
