# Supplementary material for: Fetal Head Growth and Head Circumference at Birth in Children of Women with Psychotic Disorders and Population-Based Controls
Source: Schizophr Bull. 2025 Sep 28;52(2):sbaf171. doi: 10.1093/schbul/sbaf171 (PMC12996895; doi:10.1093/schbul/sbaf171)
Supplement: Supplementary_materials_sbaf171 [file supplementary_materials_sbaf171.docx]

**Supplementary Material: Fetal head growth and head circumference at birth in offspring of women with psychotic disorders and population-based controls**

**Supplement 1: Eligibility and inclusion flow chart for clinical group and control group**


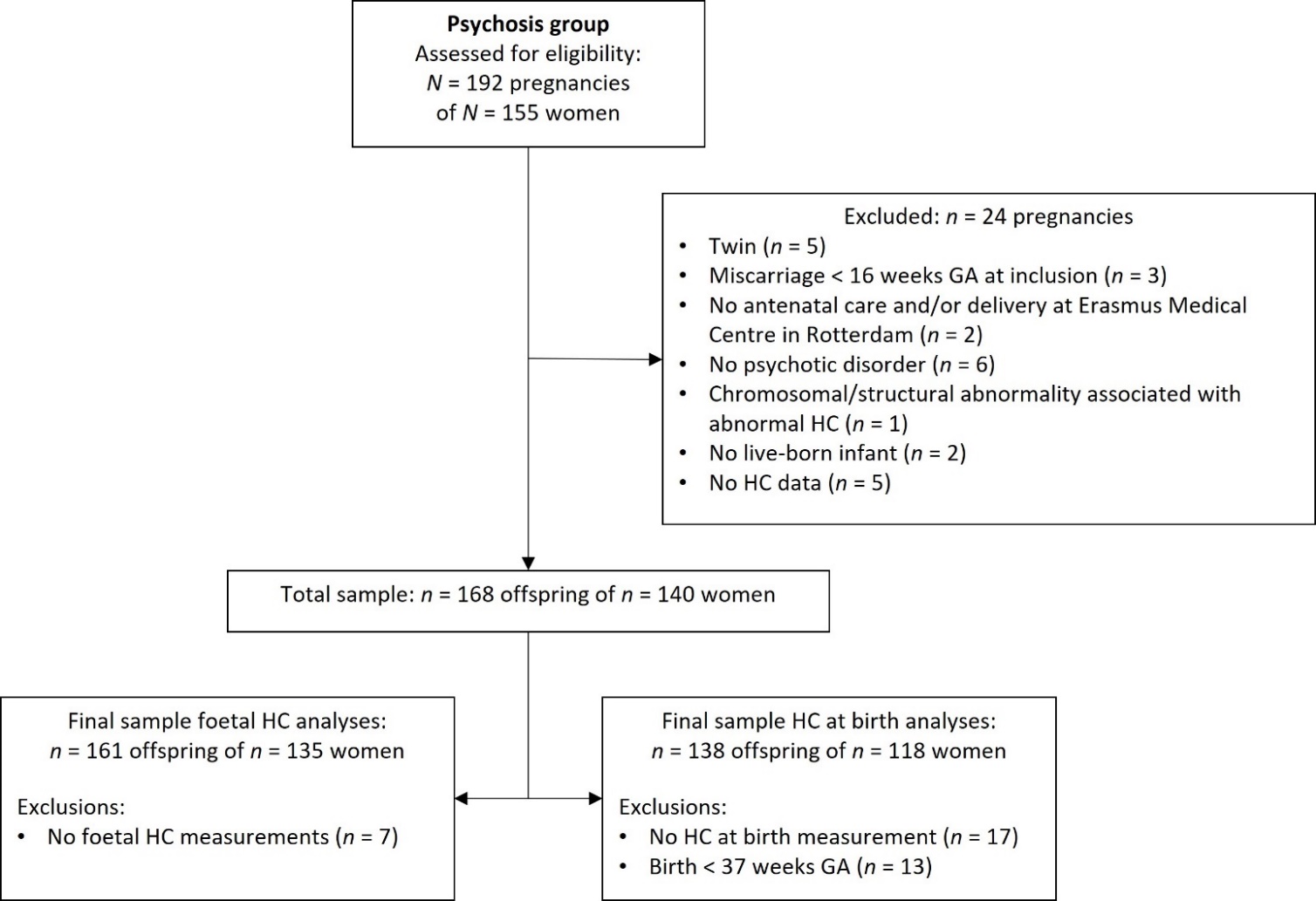


**
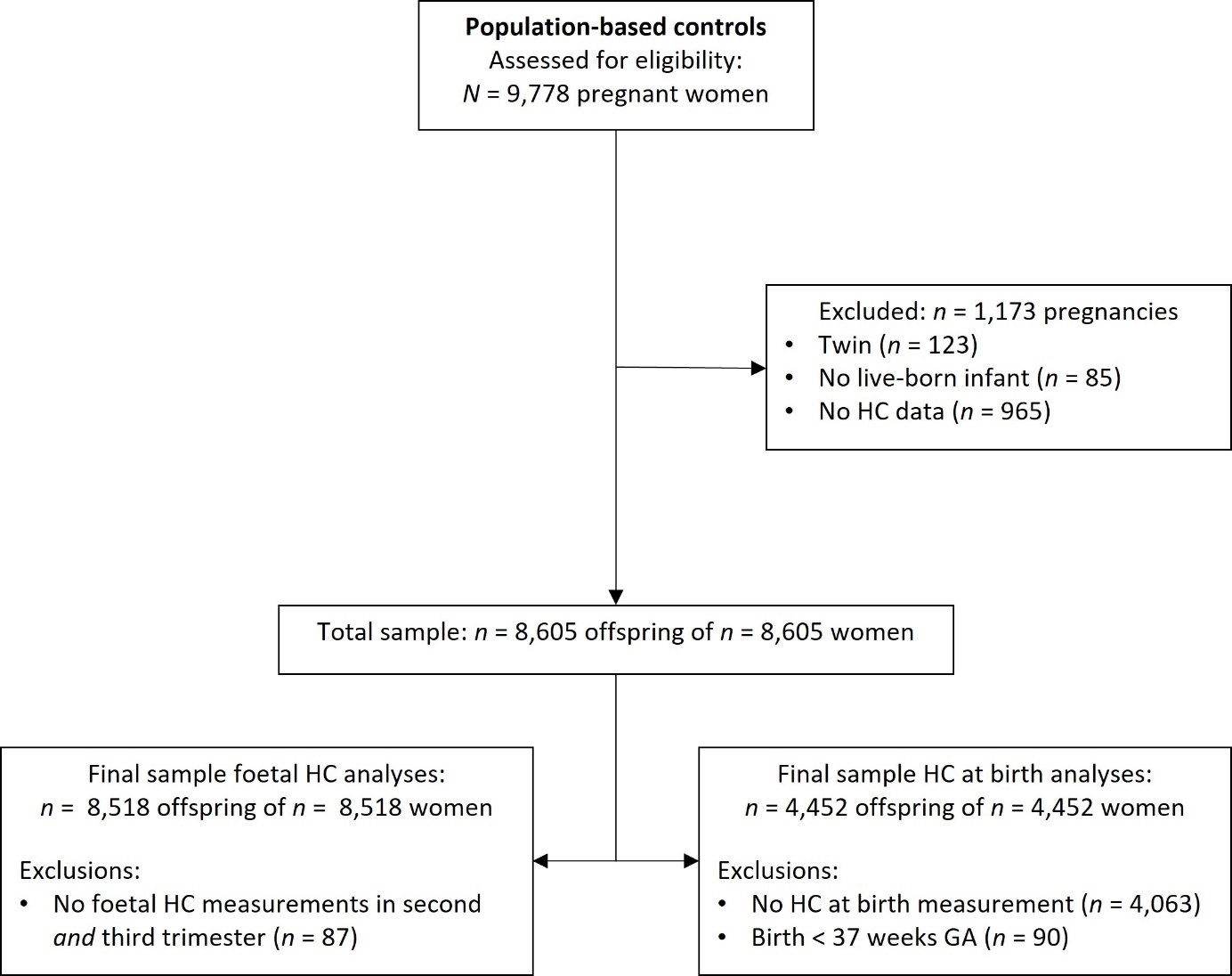
**

**Supplement 2: Including first trimester HC data in fetal analyses**

For harmonization purposes, we deviated from the preregistration and only included HC data from the second and third trimesters, as no data from the first trimester was available in the clinical group. Nonetheless, all group findings with regards to fetal HC remained stable when including first trimester data of control offspring (*n =* 6,676 observations), see Figure S1 and Table S1 for LME effect parameters.

Post-hoc analyses using pairwise comparisons of estimated marginal means with multiple comparison adjustments (Tukey’s HSD) showed that the starting point where offspring of women with a psychotic disorder started to show decreased fetal HC growth relative to control offspring was a bit earlier: from 30.8 (vs. 31) weeks GA onwards (*b* = 1.51, *SE* = 0.69, *t*(2755) = 2.18, *p* = .029).


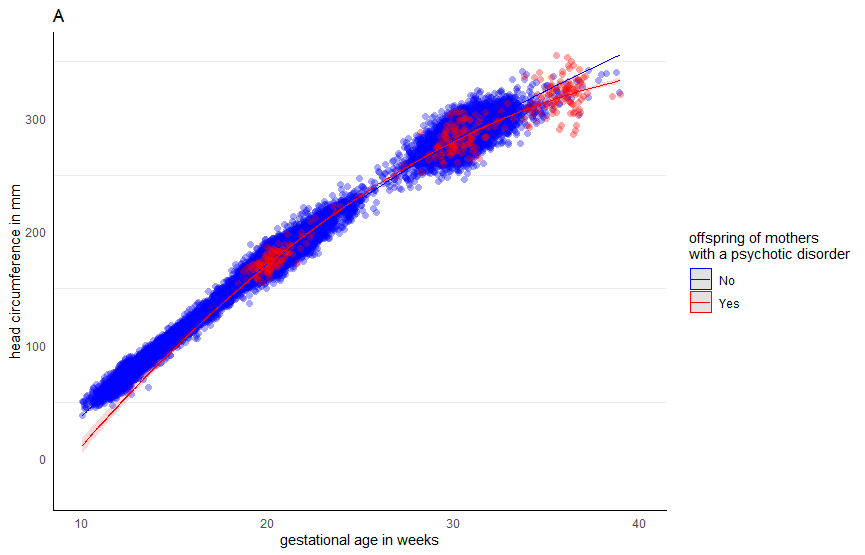


***Figure S1.*** **Differences in fetal head circumference growth between offspring of women with psychotic disorders (red) and control offspring (blue), plotted by gestational age in weeks**. Based on linear mixed effects models that were adjusted for maternal age, BMI, child’s sex, SES (based on postal code), parity, smoking, gestational diabetes, substance use, and psychotropic medication use. Shaded areas represent 2.5% and 97.5% confidence intervals (i.e. ±1.96*standard error).

**Table S1. Linear mixed effects model results of fetal head growth differences between offspring of women with a psychotic disorder and control offspring, when excluding offspring of women who self-reported having a psychiatric disorder and/or used medication within the control group (subgroup i).**

| **Effect** | **Estimate b** | **Standard Error** | **Statistic t (df)** | **p-value** |
| --- | --- | --- | --- | --- |
| Intercept | -125.0 | 0.74 | -169.7 (21679.5) | **<.001***** |
| Group | -78.7 | 8.92 | -8.82 (22170.8) | **<.001***** |
| Gestational age | 17.2 | 0.05 | 331.8 (22176.6) | **<.001***** |
| Gestational age^2^ | -0.13 | 0.00 | -111.1 (22176.6) | **<.001***** |
| Gestational age*Group | 6.51 | 0.67 | 9.68 (22175.6) | **<.001***** |
| Gestational age^2^*Group | -0.13 | 0.01 | -10.7 (22175.6) | **<.001***** |

*Note.* Findings from linear mixed effects (LME) model testing for a significant difference in the nonlinear trajectory of fetal head growth between offspring of women with a psychotic disorder and control offspring. Pooled LME model effect parameters of fetal head circumference predicted by group (reference = control group), gestational age, gestational age^2^, and their interactions are shown. The model was adjusted for child’s sex, maternal age, BMI, SES (based on postal code), parity, smoking, gestational diabetes, substance use, and psychotropic medication use.

**Supplement 3**

**Table S2. Linear mixed effects model results of fetal head growth differences between offspring of women with a psychotic disorder and control offspring, when only including main effects**.

| **Effect** | **Estimate b** | **Standard Error** | **Statistic t (df)** | **p-value** |
| --- | --- | --- | --- | --- |
| Intercept | -177.0 | 2.71 | -65.4 (16531.8) | **<.001***** |
| Group | -2.20 | 0.65 | -3.37 (8153.0) | **<.001***** |
| Gestational age | 21.3 | 0.21 | 99.6 (16548.5) | **<.001***** |
| Gestational age^2^ | -0.21 | 0.00 | -49.6 (16548.4) | **<.001***** |
| Child’s sex | 2.74 | 0.14 | 19.1 (16513.5) | **<.001***** |
| Maternal age | 0.06 | 0.01 | 4.40 (16398.9) | **<.001***** |
| Maternal BMI | 0.12 | 0.02 | 7.12 (13380.2) | **<.001***** |
| Parity | -0.12 | 0.15 | -0.76 (14389.1) | .449 |
| Smoking | -1.19 | 0.20 | -6.03 (2305.8) | **<.001***** |
| Gestational diabetes | 1.47 | 0.66 | 2.23 (5910.0) | **.026*** |
| Substance use | 0.27 | 0.26 | 1.04 (3124.1) | .297 |
| Psychotropic medication use | -0.16 | 0.43 | -0.38 (1534.5) | .704 |
| Low SES | -0.43 | 0.18 | -2.34 (16442.0) | **.020*** |

*Note.* Findings from linear mixed effects (LME) model testing for a significant difference in the nonlinear trajectory of fetal head growth between offspring of women with a psychotic disorder and control offspring. Pooled linear mixed effects model effect parameters of fetal head circumference predicted by group (reference = control group), gestational age, and gestational age^2^, while controlling for child’s sex, maternal age, BMI, SES (based on postal code), parity, smoking, gestational diabetes, substance use, and psychotropic medication use.

**Supplement 4: Sensitivity analyses – fetal HC**

All group findings with regards to fetal HC remained stable after (i) excluding offspring of women who self-reported having a psychiatric disorder and/or used medication within the control group (control group: *n =* 5,986; clinical group: *n =* 161), see Table S3 for LME effect parameters; (ii) retaining offspring of women who self-reported having a psychiatric disorder, but excluding offspring of women who used medication within the control group (control group: *n =* 7,572; clinical group: *n =* 161), see Table S4 for LME effect parameters; (iii) retaining offspring of women who used medication, but excluding offspring of women who self-reported having a psychiatric disorder within the control group (control group: *n =* 6,547; clinical group: *n =* 161), see Table S5 for LME effect parameters; (iv) excluding offspring of women with fetal growth restriction and/or who used substances during pregnancy (control group: *n =* 5,290; clinical group: *n =* 94), see Table S6 for LME effect parameters.

Post-hoc analyses using pairwise comparisons of estimated marginal means with multiple comparison adjustments (Tukey’s HSD) showed that only the starting point where offspring of women with a psychotic disorder started to show decreased fetal HC growth relative to control offspring varied slightly: earlier GA for subgroups i-iii, and later GA for subgroup iv. That is, for subgroup i: from 30.6 (vs. 31) weeks GA onwards (*b* = 2.72, *SE* = 1.35, *t*(2025) = 2.01, *p* = .045); for subgroup ii: from 30.7 (vs. 31) weeks GA onwards (*b* = 2.68, *SE* = 1.34, *t*(2279) = 2.00, *p* = .046); for subgroup iii: from 30.8 (vs. 31) weeks GA onwards (*b* = 1.66, *SE* = 0.83, *t*(2658) = 2.00, *p* = .045); for subgroup iv: from 32.5 (vs. 31) weeks GA onwards (*b* = 1.88, *SE* = 0.95, *t*(1438) = 1.98, *p* = .049).

**Table S3. Linear mixed effects model results of fetal head growth differences between offspring of women with a psychotic disorder and control offspring, when excluding offspring of women who self-reported having a psychiatric disorder and/or used medication within the control group (subgroup i).**

| **Effect** | **Estimate b** | **Standard Error** | **Statistic t (df)** | **p-value** |
| --- | --- | --- | --- | --- |
| Intercept | -165.1 | 3.66 | -45.2 (11444.0) | **<.001***** |
| Group | -42.1 | 10.1 | -4.16 (11447.3) | **<.001***** |
| Gestational age | 20.3 | 0.29 | 69.7 (11448.7) | **<.001***** |
| Gestational age^2^ | -0.19 | 0.01 | -32.8 (11448.7) | **<.001***** |
| Gestational age*Group | 3.43 | 0.76 | 4.48 (11448.1) | **<.001***** |
| Gestational age^2^*Group | -0.07 | 0.01 | -5.01 (11448.1) | **<.001***** |

*Note.* Findings from linear mixed effects (LME) model testing for a significant difference in the nonlinear trajectory of fetal head growth between offspring of women with a psychotic disorder and control offspring. Pooled LME model effect parameters of fetal head circumference predicted by group (reference = control group), gestational age, gestational age^2^, and their interactions are shown. The model was adjusted for child’s sex, maternal age, BMI, SES (based on postal code), parity, smoking, gestational diabetes, substance use, and psychotropic medication use.

**Table S4. Linear mixed effects model results of fetal head growth differences between offspring of women with a psychotic disorder and control offspring, when excluding offspring of women who used psychotropic medication within the control group (subgroup ii).**

| **Effect** | **Estimate b** | **Standard Error** | **Statistic t (df)** | **p-value** |
| --- | --- | --- | --- | --- |
| Intercept | -165.7 | 3.22 | -51.4 (14826.8) | **<.001***** |
| Group | -41.3 | 9.90 | -4.17 (14828.6) | **<.001***** |
| Gestational age | 20.3 | 0.26 | 79.3 (14829.4) | **<.001***** |
| Gestational age^2^ | -0.19 | 0.01 | -37.4 (14829.4) | **<.001***** |
| Gestational age*Group | 3.37 | 0.75 | 4.52 (14829.2) | **<.001***** |
| Gestational age^2^*Group | -0.07 | 0.01 | -5.07 (14829.2) | **<.001***** |

*Note.* Findings from linear mixed effects (LME) model testing for a significant difference in the nonlinear trajectory of fetal head growth between offspring of women with a psychotic disorder and control offspring. Pooled LME model effect parameters of fetal head circumference predicted by group (reference = control group), gestational age, gestational age^2^, and their interactions are shown. The model was adjusted for child’s sex, maternal age, BMI, SES (based on postal code), parity, smoking, gestational diabetes, substance use, and psychotropic medication use.

**Table S5. Linear mixed effects model results of fetal head growth differences between offspring of women with a psychotic disorder and control offspring, when excluding offspring of women who self-reported having a psychiatric disorder within the control group (subgroup iii).**

| **Effect** | **Estimate b** | **Standard Error** | **Statistic t (df)** | **p-value** |
| --- | --- | --- | --- | --- |
| Intercept | -166.5 | 3.41 | -48.8 (12731.1) | **<.001***** |
| Group | -39.2 | 9.94 | -3.94 (12733.6) | **<.001***** |
| Gestational age | 20.4 | 0.27 | 75.4 (12738.9) | **<.001***** |
| Gestational age^2^ | -0.19 | 0.01 | -35.8 (12738.8) | **<.001***** |
| Gestational age*Group | 3.28 | 0.76 | 4.34 (12740.6) | **<.001***** |
| Gestational age^2^*Group | -0.07 | 0.01 | -4.87 (12740.7) | **<.001***** |

*Note.* Findings from linear mixed effects (LME) model testing for a significant difference in the nonlinear trajectory of fetal head growth between offspring of women with a psychotic disorder and control offspring. Pooled LME model effect parameters of fetal head circumference predicted by group (reference = control group), gestational age, gestational age^2^, and their interactions are shown. The model was adjusted for child’s sex, maternal age, BMI, SES (based on postal code), parity, smoking, gestational diabetes, substance use, and psychotropic medication use.

**Table S6. Linear mixed effects model results of fetal head growth differences between offspring of women with a psychotic disorder and control offspring, when excluding offspring with fetal growth restriction and/or offspring of women who used substances (i.e., alcohol, hard drugs, cannabis, cigarettes) during pregnancy (subgroup iv).**

| **Effect** | **Estimate b** | **Standard Error** | **Statistic t (df)** | **p-value** |
| --- | --- | --- | --- | --- |
| Intercept | -169.8 | 3.76 | -45.1 (10363.5) | **<.001***** |
| Group | -42.1 | 12.0 | -3.50 (10363.0) | **<.001***** |
| Gestational age | 20.6 | 0.30 | 69.1 (10363.9) | **<.001***** |
| Gestational age^2^ | -0.19 | 0.01 | -33.1 (10363.9) | **<.001***** |
| Gestational age*Group | 3.41 | 0.91 | 3.76 (10363.8) | **<.001***** |
| Gestational age^2^*Group | -0.07 | 0.02 | -4.07 (10363.9) | **<.001***** |

*Note.* Findings from linear mixed effects (LME) model testing for a significant difference in the nonlinear trajectory of fetal head growth between offspring of women with a psychotic disorder and control offspring. Pooled LME model effect parameters of fetal head circumference predicted by group (reference = control group), gestational age, gestational age^2^, and their interactions are shown. The model was adjusted for child’s sex, maternal age, BMI, SES (based on postal code), parity, gestational diabetes, and psychotropic medication use.

**Supplement 5: Sensitivity analyses – HC at birth**

At birth, we found a significant effect of group on HC (*b* = 0.51, *SE* = 0.23, *t*(2905.5) = 2.18, *p* = .029) when rerunning analyses with subgroup iv (i.e., excluding offspring of women with fetal growth restriction and/or offspring of women who used substances during pregnancy; control group: *n =* 2,930; clinical group: *n =* 87), indicating that HC at birth was *larger* in offspring of women with psychotic disorders compared to control offspring. The other two group findings remained stable: HC/birthweight ratio (*b* = 0.00, *SE* = 0.00, *t*(2965.5) = 3.25, *p* <.001); birthweight (*b* = -123.0, *SE* = 62.0, *t*(2971.4) = -1.98, *p* = .047). Furthermore, findings did not change when rerunning our analyses with subgroup iii (i.e., retaining offspring of women who used medication, but excluding offspring of women who self-reported having a psychiatric disorder within the control group (control group: *n =* 3,387; clinical group: *n =* 138): HC at birth (*b* = 0.15, *SE* = 0.21, *t*(1910.7) = 0.71, *p* = .477); HC/birthweight ratio (*b* = 0.00, *SE* = 0.00, *t*(2269.7) = 2.70, *p* = .007); birthweight (*b* = -109.3, *SE* = 55.3, *t*(2345.3) = -1.97, *p* = .048).

However, group findings disappeared when rerunning analyses with subgroup i (i.e., excluding offspring of women who self-reported having a psychiatric disorder and/or used medication within the control group (control group: *n =* 3,032; clinical group: *n =* 138): HC at birth (*b* = 0.21, *SE* = 0.32, *t*(3144.3) = 0.64, *p* = .524); HC/birthweight ratio (*b* = 0.00, *SE* = 0.00, *t*(3131.1) = 1.56, *p* = .119); birthweight (*b* = -91.8, *SE* = 84.8, *t*(3127.0) = -1.08, *p* = .279) and subgroup ii (i.e., retaining offspring of women who self-reported having a psychiatric disorder, but excluding offspring of women who used medication within the control group (control group: *n =* 3,980; clinical group: *n =* 138): HC at birth (*b* = 0.17, *SE* = 0.32, *t*(4087.2) = 0.53, *p* = .594); HC/birthweight ratio (*b* = 0.00, *SE* = 0.00, *t*(4065.7) = 1.64, *p* = .102); birthweight (*b* = -99.6, *SE* = 83.3, *t*(4061.7) = -1.19, *p* = .232).

**Supplement 6: Sex specific effects on HC**

## *Fetal HC growth trajectories in male and female offspring*

To investigate sex specific effects on HC, we ran separate analyses for male (control group: *n* = 4,301; clinical group: *n* = 75) and female offspring (control group: *n* = 4,215; clinical group: *n* = 85). Using GAMM, we observed a non-linear increase in male fetal head growth for both the control group (e.d.f. = 1.999, *F* = 189430, *p* < .001) (Figure S2A) and the clinical group (e.d.f. = 1.992, *F* = 4742, *p* < .001) (Figure S2B), as well as in female fetal head growth: control group (e.d.f. = 1.999, *F* = 190849, *p* < .001) (Figure S3A), control group (e.d.f. = 1.994, *F* = 5645, *p* < .001) (Figure S3B). To compare these trajectories with each other, we plotted a difference curve for each sex. The difference curve showed a positive value (a less pronounced increase for offspring of women with a psychotic disorder relative to control offspring) from 32.0 weeks GA onwards in male offspring (Figure S2C), and from 30.7 weeks GA onwards in female offspring (Figure S3C). Furthermore, between 21.8 and 25.6 weeks GA high-risk male offspring showed increased fetal HC growth compared to control male offspring, see Figure S2C.


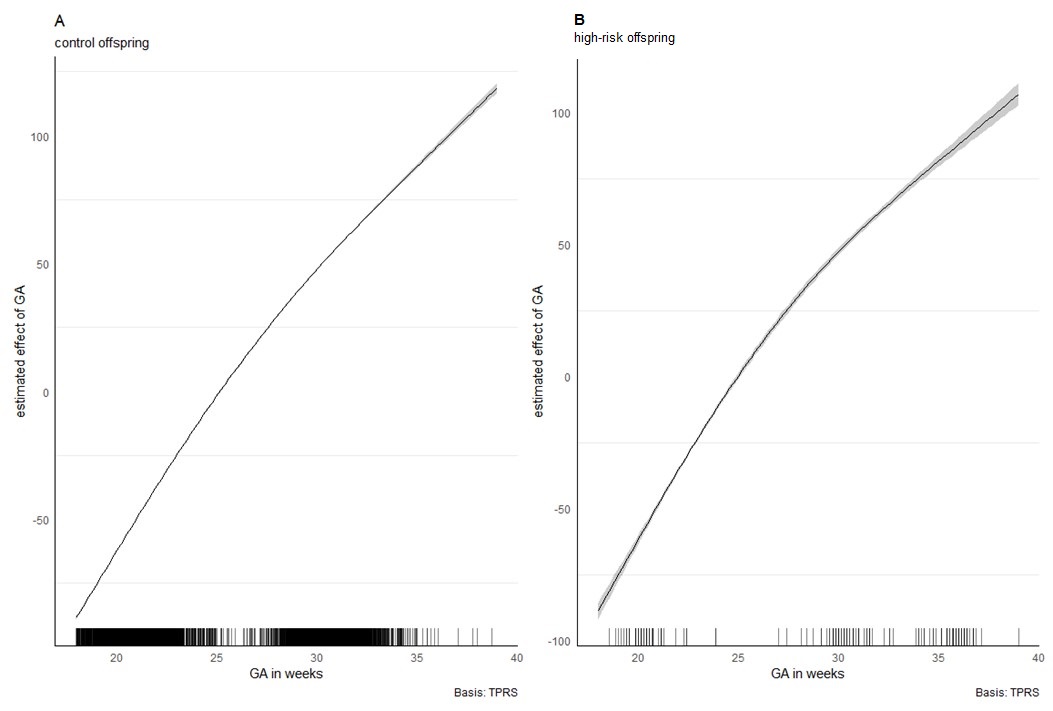


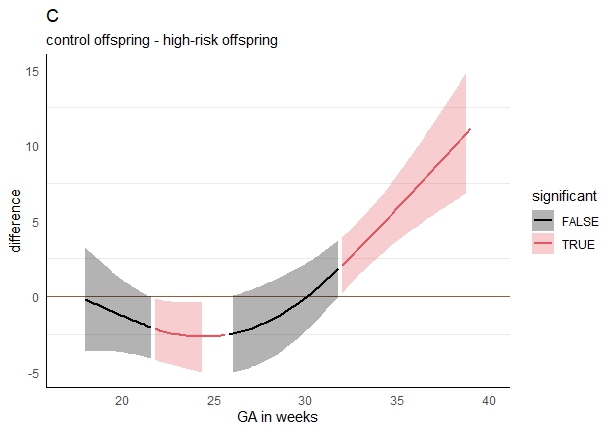


***Figure S2.* Fetal head circumference growth trajectories using GAMM in male offspring.** Trajectories estimating the effect of gestational age (GA) in weeks for control male offspring (**A**) and male offspring of women with a psychotic disorder (**B**) separately. The y-axis displays the GAM-estimated additive effect of GA in weeks. Black markings on the x-axis show individual GA data points. **C:** Difference curve graphically showing the differences between the two trajectories in panels A and B (i.e., subtracting the estimated effects of GA: control male offspring – high-risk male offspring). Trajectories are considered to be significantly different if the confidence interval does not include zero (i.e., red part of curve: from 32.0 weeks GA onwards; between 21.8 and 25.6 weeks GA high-risk offspring showed increased fetal HC growth compared to control offspring).


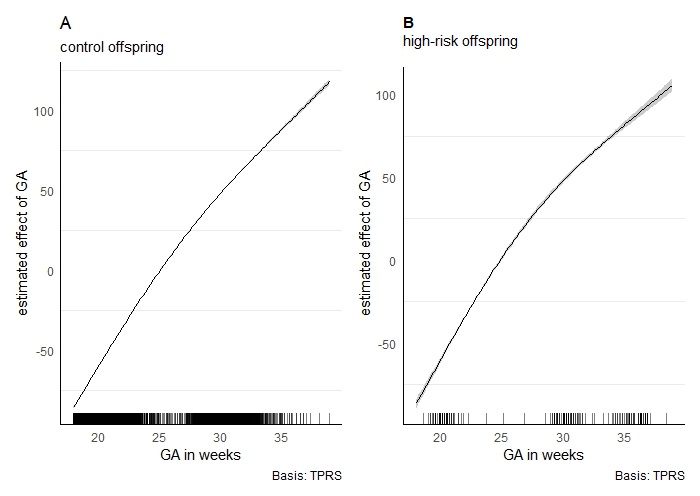


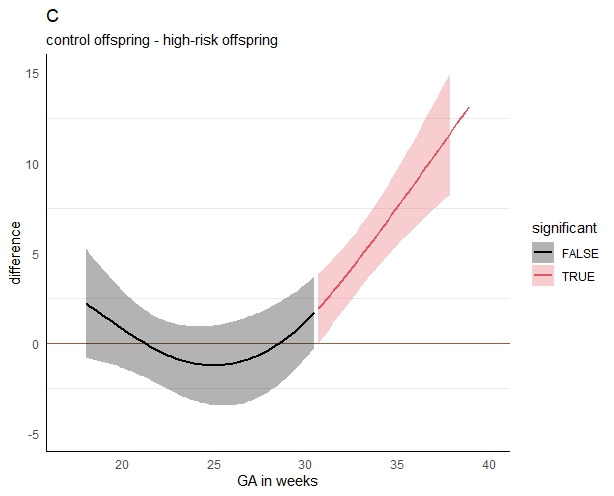


***Figure S3.* Fetal head circumference growth trajectories using GAMM in female offspring.** Trajectories estimating the effect of gestational age (GA) in weeks for control female offspring (**A**) and female offspring of women with a psychotic disorder (**B**) separately. The y-axis displays the GAM-estimated additive effect of GA in weeks. Black markings on the x-axis show individual GA data points. **C:** Difference curve graphically showing the differences between the two trajectories in panels A and B (i.e., subtracting the estimated effects of GA: control female offspring – high-risk female offspring). Trajectories are considered to be significantly different if the confidence interval does not include zero (i.e., red part of curve: from 30.7 weeks onwards).

To test these differences statistically, we used LME modelling, and found a significant difference in the nonlinear trajectory of fetal HC growth between male groups, i.e., a significant interaction between group and GA^2^ (*b* = -0.05, *SE* = 0.02, *t*(8330.4) = -2.77, *p* = .006), see Figure S4 and Table S7 for effect parameters, as well as between female groups (*b* = -0.08, *SE* = 0.02, *t*(8195.1) = -4.43, *p* < .001), see Figure S5 and Table S8 for effect parameters. Post-hoc analyses using pairwise comparisons of estimated marginal means with multiple comparison adjustments (Tukey’s HSD) showed that from 32.1 weeks GA onwards, male offspring of women with a psychotic disorder showed decreased fetal HC growth relative to control male offspring (*b* = 2.21 *SE* = 1.08, *t*(3319) = 2.04, *p* = .042). For female offspring, decreased fetal HC growth in the clinical group relative to the control group was observed from 31.1 weeks GA onwards (*b* = 2.07 *SE* = 1.04, *t*(1007) = 1.99, *p* = .047).

## *HC at birth findings in male and female offspring*

To investigate sex specific effects on HC at birth, we ran separate analyses for male (control group: *n* = 2,237; clinical group: *n* = 65) and female offspring (control group: *n* = 2,213; clinical group: *n* = 73). We found no significant difference in head circumference between male groups (*b* = 0.42, *SE* = 0.26, *t*(1994.8) = 1.62, *p* = .104) or female groups (*b* = 0.11, *SE* = 0.24, *t*(2052.8) = 0.43, *p* = .661). However, we found a significant effect of group on HC/birthweight ratio both for males (*b* = 0.00, *SE* = 0.00, *t*(1814.4) = 2.57, *p* = .010) and females (*b* = 0.00, *SE* = 0.00, *t*(1935.8) = 3.21, *p* = .001), as well as a significant effect on birthweight for females (*b* = -155.1, *SE* = 62.4, *t*(2060.1) = -2.87, *p* = .013), but not for males (*b* = -110.6, *SE* = 69.6, *t*(1968.7) = -1.59, *p* = .112).


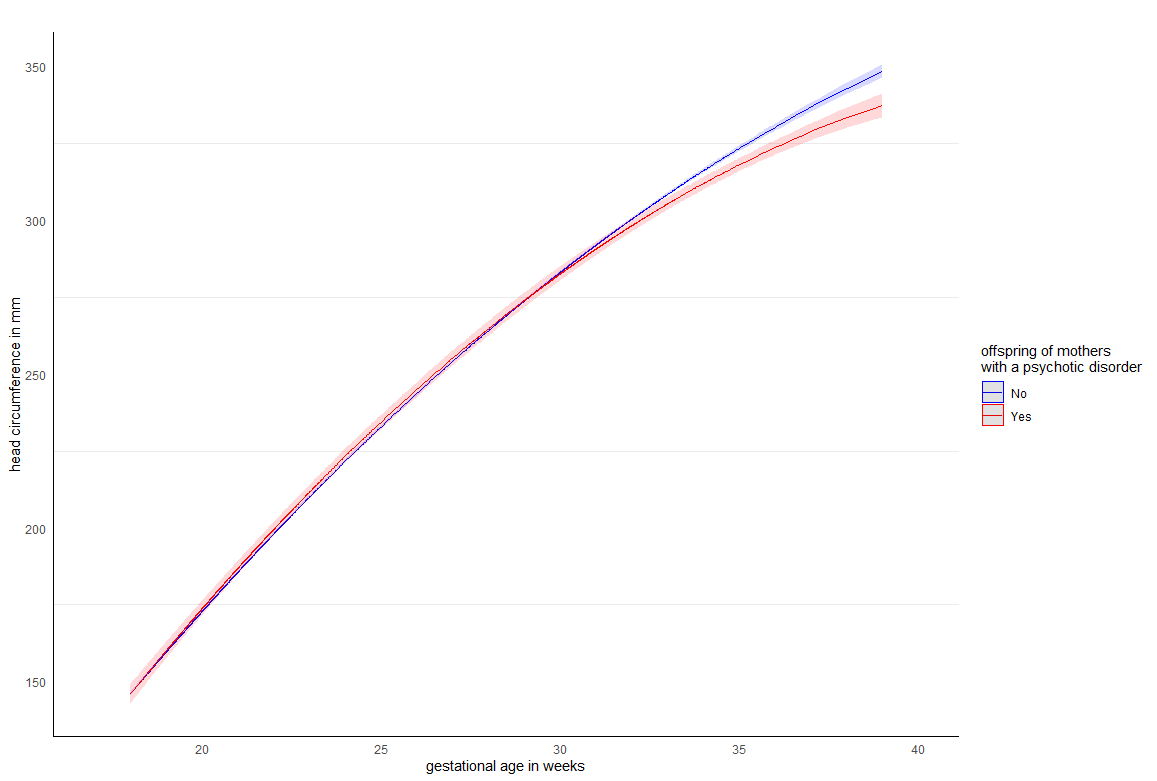


***Figure S4.*** **Differences in fetal head circumference growth between male offspring of women with psychotic disorders (red) and control male offspring (blue), plotted by gestational age in weeks**. Based on linear mixed effects models that were adjusted for maternal age, BMI, SES (based on postal code), parity, smoking, gestational diabetes, substance use, and psychotropic medication use. Shaded areas represent 2.5% and 97.5% confidence intervals (i.e. ±1.96*standard error).

**Table S7. Linear mixed effects model results of fetal head growth differences between male offspring of women with a psychotic disorder and control male offspring.**

| **Effect** | **Estimate b** | **Standard Error** | **Statistic t (df)** | **p-value** |
| --- | --- | --- | --- | --- |
| Intercept | -171.2 | 4.35 | -39.4 (8325.6) | **<.001***** |
| Group | -28.8 | 14.4 | -2.01 (8330.8) | **.045*** |
| Gestational age | 20.9 | 0.35 | 60.5 (8329.3) | **<.001***** |
| Gestational age^2^ | -0.19 | 0.01 | -29.2 (8329.3) | **<.001***** |
| Gestational age*Group | 2.59 | 1.09 | 2.38 (8330.5) | **.017*** |
| Gestational age^2^*Group | -0.05 | 0.02 | -2.77 (8330.4) | **.006**** |

*Note.* Findings from linear mixed effects (LME) model testing for a significant difference in the nonlinear trajectory of fetal head growth between male offspring of women with a psychotic disorder and control male offspring. Pooled LME model effect parameters of fetal head circumference predicted by group (reference = control group), gestational age, gestational age^2^, and their interactions are shown. The model was adjusted for age, BMI, SES (based on postal code), parity, smoking, gestational diabetes, substance use, and psychotropic medication use.


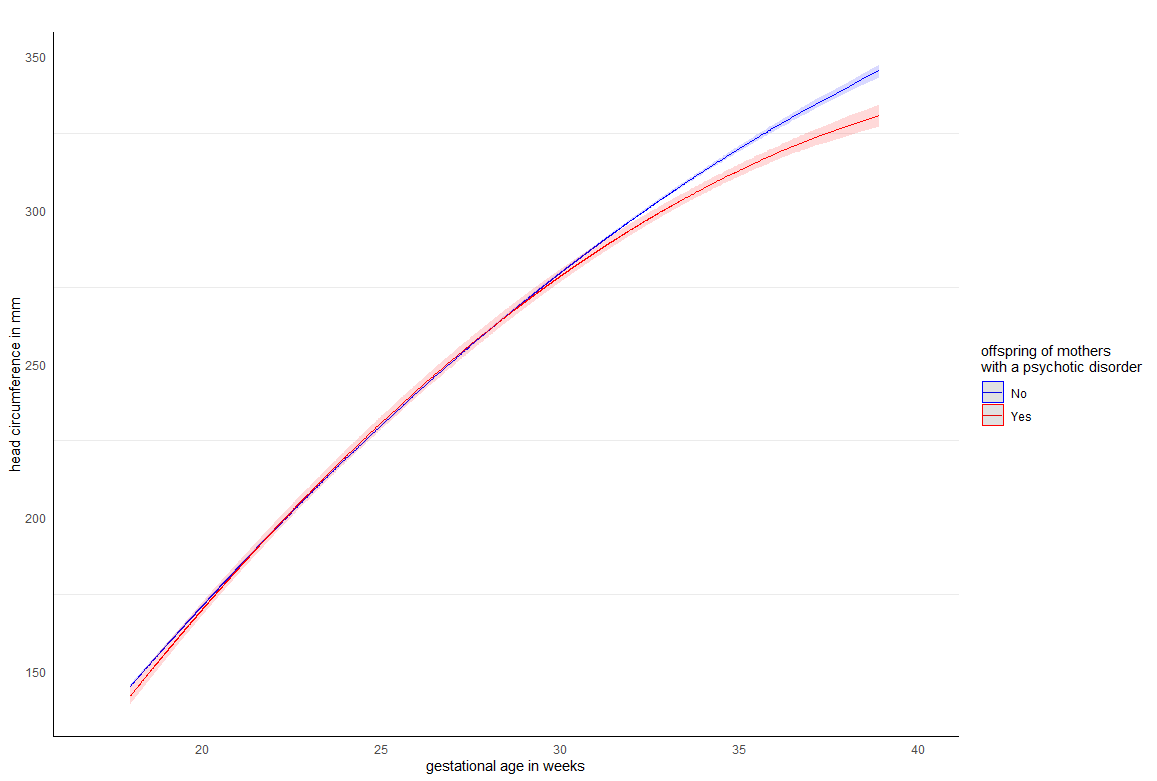


***Figure S5.*** **Differences in fetal head circumference growth between female offspring of women with psychotic disorders (red) and control female offspring (blue), plotted by gestational age in weeks**. Based on linear mixed effects models that were adjusted for maternal age, BMI, SES (based on postal code), parity, smoking, gestational diabetes, substance use, and psychotropic medication use. Shaded areas represent 2.5% and 97.5% confidence intervals (i.e. ±1.96*standard error).

**Table S8. Linear mixed effects model results of fetal head growth differences between female offspring of women with a psychotic disorder and control female offspring.**

| **Effect** | **Estimate b** | **Standard Error** | **Statistic t (df)** | **p-value** |
| --- | --- | --- | --- | --- |
| Intercept | -160.2 | 4.15 | -38.6 (8190.6) | **<.001***** |
| Group | -49.6 | 13.1 | -3.78 (8193.3) | **<.001***** |
| Gestational age | 20.0 | 0.33 | 60.5 (8193.4) | **<.001***** |
| Gestational age^2^ | -0.18 | 0.01 | -28.3 (8193.4) | **<.001***** |
| Gestational age*Group | 4.03 | 1.00 | 4.05 (8195.0) | **<.001***** |
| Gestational age^2^*Group | -0.08 | 0.02 | -4.43 (8195.0) | **<.001***** |

*Note.* Findings from linear mixed effects (LME) model testing for a significant difference in the nonlinear trajectory of fetal head growth between female offspring of women with a psychotic disorder and control female offspring. Pooled LME model effect parameters of fetal head circumference predicted by group (reference = control group), gestational age, gestational age^2^, and their interactions are shown. The model was adjusted for age, BMI, SES (based on postal code), parity, smoking, gestational diabetes, substance use, and psychotropic medication use.
